# Dengue induces iNOS expression and nitric oxide synthesis in platelets through IL-1R

**DOI:** 10.3389/fimmu.2022.1029213

**Published:** 2022-12-07

**Authors:** Mariana Brandi Mendonça Pinheiro, Stephane Vicente Rozini, Anna Cecíllia Quirino-Teixeira, Giselle Barbosa-Lima, Juliana F. Lopes, Carolina Q. Sacramento, Fernando A. Bozza, Patrícia T. Bozza, Eugenio D. Hottz

**Affiliations:** ^1^ Laboratory of Immunothrombosis, Department of Biochemistry, Institute of Biological Sciences, Federal University of Juiz de Fora, Juiz de Fora, Brazil; ^2^ Laboratory of Immunopharmacology, Oswaldo Cruz Institute, Oswaldo Cruz Foundation (FIOCRUZ), Rio de Janeiro, Brazil; ^3^ National Institute for Science and Technology on Innovation in Diseases of Neglected Populations (INCT/IDPN), Center for Technological Development in Health (CDTS), Fiocruz, Rio de Janeiro, Brazil; ^4^ National Institute of Infectious Disease Evandro Chagas, Oswaldo Cruz Foundation, Rio de Janeiro, Brazil; ^5^ D’Or Institute for Research and Education, Rio de Janeiro, Brazil

**Keywords:** platelets, nitric oxide, iNOS, IL-1R, dengue

## Abstract

**Introduction:**

Dengue is an arthropod-born disease caused by dengue virus (DENV), that may manifest as a mild illness or severe form, characterized by hemorrhagic fever and shock. Nitric oxide (NO) is a vasodilator signaling molecule and an inhibitor of platelet aggregation known to be increased in platelets from dengue patients. However, the mechanisms underlying NO synthesis by platelets during dengue are not yet elucidated. IL-1β is a pro-inflammatory cytokine able to induce iNOS expression in leukocytes and present in dengue patients at high levels. Nevertheless, the role of IL-1β in platelet activation, especially regarding iNOS expression, are not clear.

**Methods:**

We prospectively followed a cohort of 28 dengue-infected patients to study NO synthesis in platelets and its relationship with disease outcomes. We used in vitro infection and stimulation models to gain insights on the mechanisms.

**Results and Discussion:**

We confirmed that platelets from dengue patients express iNOS and produce higher levels of NO during the acute phase compared to healthy volunteers, returning to normal levels after recovery. Platelet NO production during acute dengue infection was associated with the presence of warning signs, hypoalbuminemia and hemorrhagic manifestations, suggesting a role in dengue pathophysiology. By investigating the mechanisms, we evidenced increased iNOS expression in platelets stimulated with dengue patients´ plasma, indicating induction by circulating inflammatory mediators. We then investigated possible factors able to induce platelet iNOS expression and observed higher levels of IL-1β in plasma from patients with dengue, which were correlated with NO production by platelets. Since platelets can synthesize and respond to IL-1β, we investigated whether IL-1β induces iNOS expression and NO synthesis in platelets. We observed that recombinant human IL-1β enhanced iNOS expression and dose-dependently increased NO synthesis by platelets. Finally, platelet infection with DENV in vitro induced iNOS expression and NO production, besides the secretion of both IL-1α and IL-1β. Importantly, treatment with IL-1 receptor antagonist or a combination of anti-IL-1α and anti-IL-1β antibodies prevented DENV-induced iNOS expression and NO synthesis. Our data show that DENV induces iNOS expression and NO production in platelets through mechanisms depending on IL-1 receptor signaling.

## Introduction

Dengue is a disease caused by dengue virus (DENV) and transmitted by *Aedes aegypti* mosquitoes. The disease affects mainly the tropical and subtropical regions and has become a serious health issue worldwide with almost 400 million new infections each year (1, 2). Dengue usually manifests as a mild illness characterized by a self-limiting infection. It might also present with warning signs including mucosal bleeding, fluid accumulation and rapid thrombocytopenia that can evolve to severe dengue, characterized by plasma leakage, hemodynamic instability, hemorrhagic fever, and shock (3).After the incubation period, the disease starts with the initial febrile phase with typical symptoms including high-grade fever, dehydration, and increased hematocrit. After the defervescence, patients may follow with a critical phase characterized by increased vascular permeability and plasma leakage that might lead to increased risk of severe dengue. The complications associated with severe dengue include severe bleeding, shock, and organ impairment, that may lead to death (3, 4).

Inflammation plays a major role in dengue pathogenesis and disease severity. It has been demonstrated that the upregulation of inflammatory mediators that synergize to amplify the inflammatory response contributes to vascular dysfunction and organ impairment (5, 6). Cytokines as IL-1β, IFN-γ, and TNF-α are elevated in severe dengue patients and associate with the degree of thrombocytopenia, hemodynamic instability, and disease severity (7). Among these cytokines, IL-1β is a potent pro-inflammatory cytokine related to the induction of fever and a trigger of nitric oxide (NO) production in many cells (8–11). NO is a vasodilator signalling molecule regulating vascular function, cell recruitment, and platelet aggregation. It is generated upon oxidation of L-arginine, catalyzed by nitric oxide synthase (NOS) enzymes and it is rapidly converted into nitrite and nitrate (12, 13). Enhanced NO bioavailability and nitrite levels are associated with vascular dysfunction and hemorrhage in dengue patients, and increased L-arginine transport and NOS activity in platelets have been associated with platelet dysfunction in dengue (14–17).

Platelet dysfunction and thrombocytopenia are hallmarks of dengue. Platelets are known mainly by their role in thrombosis and hemostasis, but they also play important roles in modulating immune response through mechanisms involving interaction with leukocytes and secretion of inflammatory mediators (18). Platelet secretion involves the translocation of granules containing stored factors such as cytokines and chemokines, and also newly synthesized mediators such as eicosanoids and NO (19–21). Platelets secrete NO through the expression of different NOS enzymes, including endothelial (eNOS) and inducible (iNOS) isoforms (12, 15). Besides immune and inflammatory response, platelets also play important roles in maintaining vascular stability and in pathological vascular inflammation and dysfunction (22–25). During dengue, patients present increased platelet activation and emerging evidence highlights the participation of platelets in dengue pathogenesis, including in inflammatory amplification and increased vascular permeability (18, 22, 25–31). However, the mechanism underlying platelet NO synthesis and its contributions to dengue pathogenesis remains poorly elucidated. In this study, we aimed to evaluate the mechanisms of iNOS expression and NO production in platelets during dengue.

## Material and methods

### Human subjects

We prospectively followed a cohort of 28 serologically and/or molecularly confirmed DENV-infected patients from the Instituto Nacional de Infectologia Evandro Chagas (INI), Fundação Oswaldo Cruz (Fiocruz), Rio de Janeiro, Brazil, during the dengue outbreaks of 2011-2013. Peripheral vein blood samples were obtained from all patients during the acute phase. The average day of sample collection after the onset of illness was 5 ± 1.9 days and the day of defervescence was 6 ± 1.8 days. All patients were followed until the discharge and a second sample was obtained from 12 patients at the recovery phase. The cohort was comprised of patients with mild to severe dengue. Specifically, 18 (64,3%) patients evolved with warning signs, of which 3 (10,7%) progressed to severe dengue syndrome. The levels of immunoglobulin M (IgM) and immunoglobulin G (IgG) specific to DENV E protein were measured using a standard capture enzyme-linked immunosorbent assay (ELISA) kit according to the manufacturer’s instructions (E-Den01M and E-Den01G; PanBio) and the IgM/IgG antibody ratio > 1.2 was used to distinguish between primary and secondary infections as previously reported (32, 33) and shown in **Table 1**. Virus typing and quantification were performed using reverse transcription polymerase chain reaction (RT-PCR) as previously described (34, 35). All patients had dengue confirmed diagnostic through RT-PCR, IgM and/or nonstructural protein 1 (NS1) positivity in blood. Peripheral vein blood was also collected from 23 age- and sex-matched healthy subjects that were included in parallel to dengue patients. The characteristics of dengue patients and control participants are presented in **Table 1**. The study protocol was approved by the Institutional Review Board (Instituto de Pesquisas Clínicas Evandro Chagas #016/2010), and the experiments were performed in compliance with this protocol. Written informed consent was obtained from all volunteers prior to any study-related procedure, according to the Declaration of Helsinki.

**Table 1:** Characteristics of dengue-infected patients and healthy volunteers.

	Control (23)	Dengue (28)	Reference values
Age, years	27 (26–35)	36 (22 – 44)	–
Gender, male	12 (52%)	14 (50%)	–
Day of sampling after symptoms onset	–	5 (4 – 6)	–
Day of defervescence	–	6 (5 – 7)	–
Platelet count, x1,000/mm^3^	–	95 (42 – 130)	150 – 450
Hematocrit, %	–	41 (39 – 45)	36 – 52
Albumin, g/dL	–	3.4 (3.0 – 3.7)	3.6 – 5.5
TGO/AST, IU/L	–	65 (43 – 128)	15 – 37
TGP/ALT, IU/L	–	68 (47 – 105)	12 – 78
Warning Signs^1^	–	18 (64.3%)	–
Severe Dengue^2^	–	3 (10.7%)	–
Signs of increased vascular permeability^3^	–	17 (60.7%)	–
Thrombocytopenia (< 100,000/mm^3^)	–	14 (50%)	–
Hemorrhagic manifestations^4^	–	7 (25%)	–
Secondary dengue infection	–	16 (57%)	–
PCR positive (DENV-1)	0 (0%)	22 (78.5%)	–
IgM positive	0 (0%)	27 (96.4%)	–
NS1 positive	0 (0%)	5 (18%)	–
IgM positive NS1 negative	0 (0%)	23 (82%)	–
NS1 positive IgM negative	0 (0%)	1 (3.5%)	–
IgM positive NS1 positive	0 (0%)	4 (14%)	–

Data are expressed as median (interquartile range) or number (%). Laboratorial data correspond to the lower (platelet count and albumin) or the higher (hematocrit, TGO/AST, and TGP/ALT) level each patient achieved in the course of the disease. ^1^ Abdominal pain or tenderness, persistent vomiting, clinical fluid accumulation, mucosal bleeding, and/or increased hematocrit concurrent with a rapid decrease in platelet count. ^2^ Severe plasma leakage, fluid accumulation, ascites, and/or massive bleeding. ^3^ According to WHO guidelines (2009). ^4^ Increase in hematocrit >20%, hypoalbuminemia (< 3.6 g/dL), postural hypotension, ascites, and/or oliguria. ^4^Gingival bleed, vaginal bleed, gastrointestinal bleed, petechiae, and exanthema.

### Platelet isolation

Platelet isolation was performed as previously described (29). Peripheral blood samples were drawn into acid-citrate-dextrose (ACD) and centrifuged at 200 x g for 20 min. Platelet-rich plasma (PRP) was obtained and recentrifuged (500 X g, 20 min) in the presence of 100 nM of prostaglandin E_1_ (PGE_1_; Cayman 1303) to pellet the platelets. Platelet pellets were resuspended in 25 ml of PSG (5 mM PIPES, 145 mM 35 NaCl, 4 mM KCl, 50 μM Na_2_HPO_4_, 1 mM MgCl_2_·6H_2_O, 5,5 mM glucose; pH 6,8) containing 100 nM PGE_1_. The platelet suspension was centrifuged again at 500 X g for 20 min and the platelet pellet was resuspended in medium 199 (M199) containing 25 mM of HEPES to the concentration of 1 x 10^9^/mL. The platelet viability in dengue patients and healthy volunteers in the present study has been previously reported (26). The viability of platelets used for *in vitro* experiments was above 95% of live platelets.

### Platelet exposure to DENV *in vitro*

DENV2 strain 16881 was propagated in C6/36 *Aedes albopictus* cells and titrated by plaque assay, as previously described (36). Supernatant from uninfected cell cultures produced in the same conditions was used as negative control (Mock). Platelets from healthy volunteers were incubated in M199 with DENV2 in a multiplicity of infection of 1 virus per platelet (MOI = 1) or incubated with the same amount of Mock for 6 hours at 37°C. After stimuli, samples were centrifuged at 900 X g for 15 minutes for supernatant harvesting and cell preparation for flow cytometry or Western blot. To investigate the mechanisms involved in platelet iNOS expression and NO production, platelets were incubated with DENV-2 in the presence or absence of IL-1 receptor antagonist (IL-1RA), anti-IL-1α (R&D 840201) or anti-IL-1β (R&D 840168) or isotype-matched control antibody.

### Platelet *in vitro* stimulation

Platelet-poor plasma (PPP) was collected and centrifuged at 2500 x g for 20 min, aliquoted, and stored at -80°C until use. Platelets from healthy volunteers were stimulated with 10% or 100% of plasma from 3-11 dengue patients or heterologous healthy volunteers for 4 hours at 37°C. The average day of sample collection after the onset of illness in patients whose plasma was used for *ex vivo* platelet stimulation was 4 ± 2.6 days. Platelets from healthy volunteers were also stimulated with 1 to 1000 pg/ml of recombinant human IL-1β (R&D 840170) for 4 hours at 37°C. After stimuli, platelets were centrifuged at 900 x g for 15 minutes for supernatant harvesting and cell preparation for flow cytometry.

### Nitrite quantification

Washed platelets (1x10^9^/mL) isolated from seven healthy volunteers, 24 dengue-infected patients at the acute phase, and 12 recovered patients were incubated at 37°C in a 5% CO_2_ atmosphere for 4 hours. After incubation, platelets were pelleted and the supernatants were harvested. Supernatants from platelets from patients or from *in vitro* infection model were used for nitrite quantification by Griess method as previously described (37, 38). Briefly, nitrite was quantified by reaction with 0.5% sulfanilamide and 0.05% N-(1-Naphthyl)-ethylenediamine dihydrochloride, while a standard curve was prepared by serial dilution of sodium nitrite (Sigma-Aldrich). Sample absorbances after Griess reaction were determined by SpectraMax microplate reader at 595nm.

### Western blot analysis

Platelets samples were lysed (NaCl 0.15 M, Tris 10 mM pH 8, EDTA 0.1 mM, Triton X-100 1%, and protease inhibitor cocktail (ROCHE)) and protein quantification was performed using BCA kit (Thermo Scientific). Protein extracts (10 µg) from platelets from 4 patients and 4 healthy volunteers were prepared with sample buffer containing β-mercaptoethanol, separated in 10% sodium dodecyl sulfate-polyacrylamide gel electrophoresis (SDS-PAGE) and transferred into nitrocellulose membrane. The membranes were blocked with Tris-buffered saline (TBS) supplemented with 0.1% Tween 20 (TBS-T) plus 5% milk for 1 hour and incubated with primary antibodies mouse anti-iNOS (BD610333) and mouse anti-human β-actin (Sigma A1978).

### Cytokine and chemokine measurement

Cytokine and chemokine levels (TNF-α, IFN-γ, MCP-1/CCL2, RANTES/CCL5, IL-1α, and IL-1β) were quantified in dengue patient’s plasma or supernatant from *in vitro* stimulated platelets using Multiplex assay kit (Bioplex, BioRad) or ELISA kit (R&D systems) according to manufacturer’s instructions.

### Flow cytometry

Platelet NO synthesis was assessed using the fluorescent probe DAF-FM diacetate as per the manufacturer’s instructions (Invitrogen, D23844) in freshly isolated platelets from patients and healthy volunteers. Intracellular labeling of iNOS was also assessed in platelets infected with DENV *in vitro*. Platelets were fixed in PBS containing paraformaldehyde 4% for 10 min, permeabilized with PBS containing 0,1% TRITON for 10 minutes, and washed with PBS. Platelet pellet was resuspended in PBS and incubated with anti-iNOS primary antibody (NBP222119) overnight at 4°C. Platelets were washed again and incubated with PE-conjugated anti-mouse IgG (e-Bioscience 12-4012-87) for 30 min, washed again and labeled with APC-conjugated anti-CD41 antibody (BD555466). Platelets were gated according to their characteristic forward and side scattering and positivity to CD41. Platelet samples (10,000 gated events) were acquired in the flow cytometer BD FACSVerse or BD FACSCalibur and analyzed using FlowJo software.

### Statistical analysis

All statistics were performed with GraphPad Prism 7 software. The Shapiro-Wilk test was used to analyze whether numerical variables followed a normal distribution. One-way analysis of variance (ANOVA) was used to compare ≥3 groups and the locations of the differences were identified by Tukey’s *post hoc* test. Comparisons between 2 groups were performed with the Mann-Whitney U test for nonparametric distributions or the Student T-test for parametric distributions using Welch’s T-test for unpaired samples or paired T-test for *in vitro* experiments with platelets from the same donor exposed to different stimuli. The correlations were performed using Pearson’s correlation test for parametric distributions and Spearman correlation test for nonparametric distributions.

## Results

### Dengue induces iNOS expression and NO synthesis by platelets

**Figure 1:**
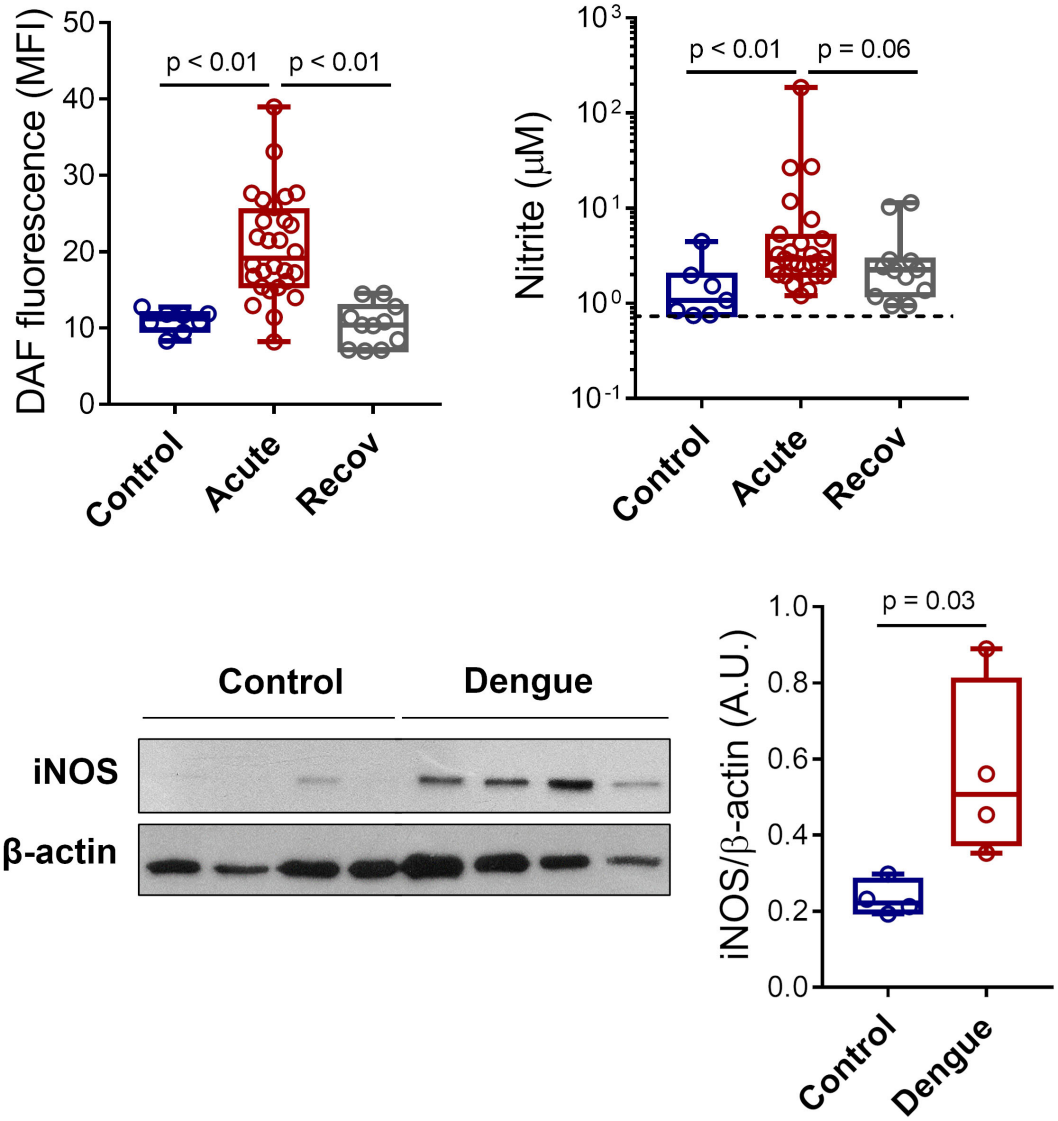
Dengue induces iNOS expression and NO synthesis in platelets. **(A)** The mean fluorescence intensity (MFI) of DAF-FM diacetate fluorescent probe indicating NO synthesis in platelets from 8 healthy volunteers (control), 28 patients with dengue at the acute phase, and 12 patients at the recovery phase. **(B)** Platelets from 7 healthy volunteers, 25 dengue-infected patients in the acute phase, and 12 patients in the recovery phase were isolated and incubated for 4 hours at 37°C and nitrite production was quantified in platelets supernatants by Griess reaction. The dotted line indicates the lower detection limit of the protocol. **(C)** Expression of iNOS in platelets lysates from 4 healthy volunteers and 4 dengue-infected patients was analyzed by western blot. β-actin was used as load control. Band densitometry of iNOS expression corrected by β-actin expression is shown (A.U., arbitrary units). Each dot represents one patient or healthy volunteer. The horizontal lines in the box plots represent the median, the box edges represent the interquartile ranges, and the whiskers indicate the minimal and maximal values in each group.

To confirm NO synthesis by platelets during Dengue infection, we quantified NO production in platelets from patients with acute dengue, healthy volunteers, and recovered patients using DAF-FM diacetate probe fluorescence (**Figure 1A**). Our data show increased NO production in platelets from patients with acute dengue compared to healthy volunteers or patients at the recovery phase (**Figure 1A**). In addition, we quantified nitrite, a product of NO metabolism, in the supernatant of platelets from patients or healthy volunteers. We found increased levels of nitrite in platelets from dengue patients at the acute phase compared to control (**Figure 1B**). We then investigated whether platelets from dengue patients express inducible NOS (iNOS or NOS2). We analyzed iNOS expression in platelets from four DENV-infected patients and four age- and sex-matched healthy volunteers by western blot. As shown in **Figure 1C**, platelets from patients with dengue express higher levels of iNOS compared to healthy volunteers.

### Platelet NO production is associated with dengue disease outcome

**Figure 2:**
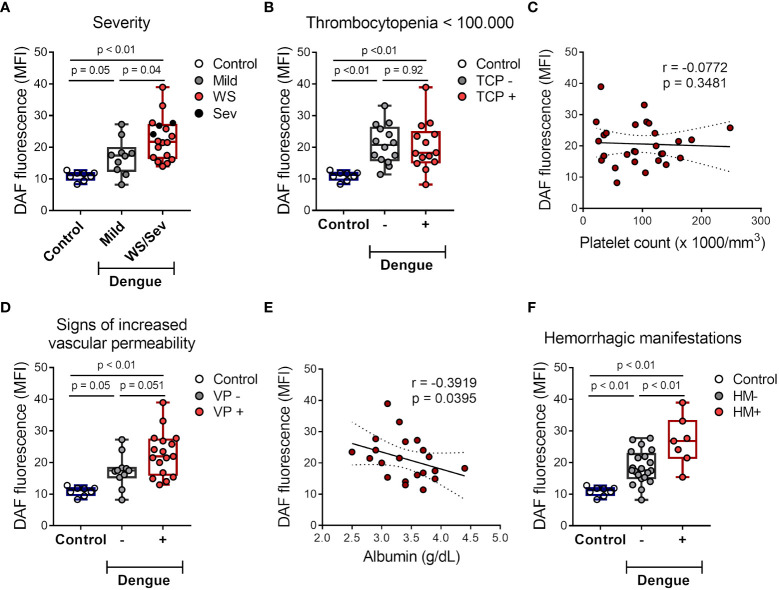
NO production by platelets from dengue-infected patients associates with disease severity. **(A)** The mean of fluorescence intensity (MFI) of DAF-FM diacetate fluorescent probe indicating NO synthesis in platelets from healthy volunteers (control) (n = 8) and patients with mild dengue (n = 10) or that evolved with warning signs (WS) or severe dengue syndrome (n = 18). **(B)** NO synthesis in platelets from control (n = 8) and dengue patients that were positive or negative for thrombocytopenia (TCP) (14 vs 14 patients, respectively). **(C)** NO synthesis in platelets was plotted against the lower platelet count in each patient, linear regression and 95 % confidence interval was traced according to the distribution of the dots. **(D–F)** NO synthesis in platelets from control (n = 8) and dengue patients that were positive or negative for clinical signs of increased vascular permeability (VP) (11 vs 17 patients, respectively) **(D)** or hemorrhagic manifestations (HM) (21 vs 7 patients, respectively) **(F)**. **(E)** NO synthesis in platelets was plotted against the lower plasma albumin levels in each patient, linear regression and 95 % confidence interval was traced according to the distribution of the dots. Each dot represents one patient or healthy volunteer. The horizontal lines in the box plots represent the median, the box edges represent the interquartile ranges, and the whiskers indicate the minimal and maximal values in each group.

Next, we investigated whether NO synthesis by platelets at the acute phase is associated with dengue disease outcomes. Platelets from patients that have evolved with warning signs and/or severe dengue syndrome showed increased NO production compared to mild illness (**Figure 2A**). Using the lowest platelet count of each patient, dengue patients were classified as thrombocytopenic (< 100,000/mm^3^) or nonthrombocytopenic. Based on this grouping, 50% of the patients were thrombocytopenic (**Table 1**). We did not find any association between platelet NO production and thrombocytopenia (**Figures 2B, C**). We have also classified the patients as positive or negative for clinical signs of increased vascular permeability according to the presence of one or more of the following signs: increased hematocrit greater than 20%, hypoalbuminemia, postural hypotension, ascites, and/or oliguria. Patients presenting clinical signs of increased vascular permeability showed a trend towards increased NO production by platelets (**Figure 2D**). Importantly, the relationship between platelet NO production and increased vascular permeability in patients was confirmed by the negative correlation between platelet NO synthesis and plasma albumin levels (**Figure 2E**), a laboratorial marker of plasma leakage. Finally, dengue patients were classified according to the presence or absence of hemorrhagic manifestations (gingival bleeding, vaginal bleeding, gastrointestinal bleeding, petechiae, and/or exanthema). We observed significantly higher NO production in platelets from patients that evolved with haemorrhagic manifestations (**Figure 2F**). Together, these results indicate that platelet NO production is higher in patients with warning signs and/or severe dengue, especially correlating with hypoalbuminemia and hemorrhage.

### Plasma from dengue patients induces iNOS expression and NO synthesis in platelets

**Figure 3:**
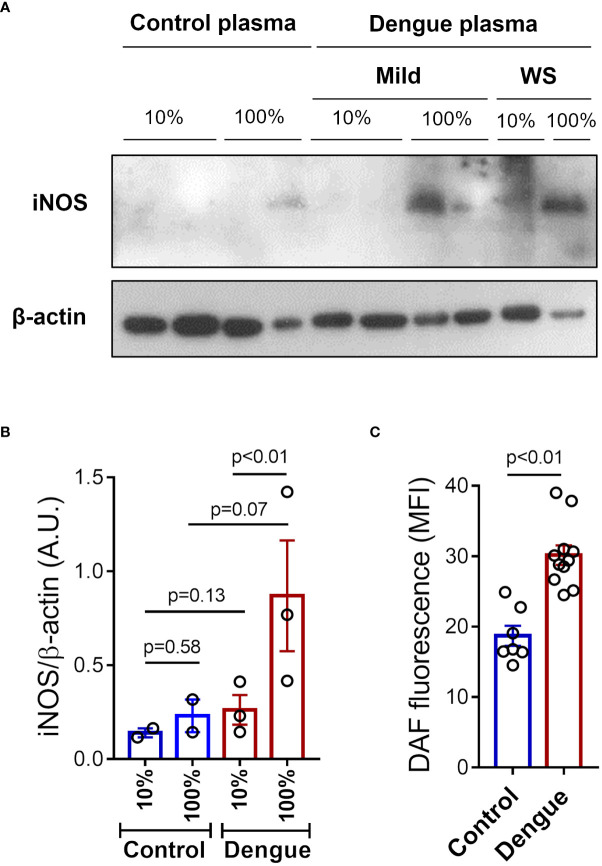
Plasma from dengue patients induces iNOS expression and NO synthesis in platelets. Platelets from healthy volunteers were incubated with 10*%* or 100% of plasma from patients with mild dengue or dengue with warning signs (WS), or heterologous healthy volunteers (control) for 4 hours at 37°C. **(A)** Expression of iNOS in platelet lysates were analyzed by western blot. β-actin was used as load control. **(B)** Band densitometry of iNOS expression corrected by β-actin expression is shown (A.U., arbitrary units). **(C)** NO production was measured by DAF in platelets incubated with 100% of plasma from control (n = 7) or dengue patients (n = 11). Each dot represents one patient or healthy volunteer. The bars represent the mean ± standard error of the mean of a representative experiment with platelets from one healthy volunteer stimulated with control or dengue patients’ plasma.

We then investigated whether inflammatory mediators in plasma from patients with dengue contribute to platelet iNOS expression and NO synthesis. Platelets from healthy volunteers were incubated with 10% or 100% plasma from dengue patients or heterologous healthy volunteers. We observed higher iNOS expression in platelets incubated with whole plasma (100%) from dengue patients compared to control (**Figures 3A, B**). In addition, we observed increased NO production in platelets exposed to 100% plasma of dengue patients compared with control plasma (**Figure 3C**). Altogether, these data show that soluble factors produced during dengue infection are at least in part responsible for the induction of iNOS expression and NO production in platelets.

### NO synthesis in platelets associates with increased IL-1β in Dengue

**Figure 4:**
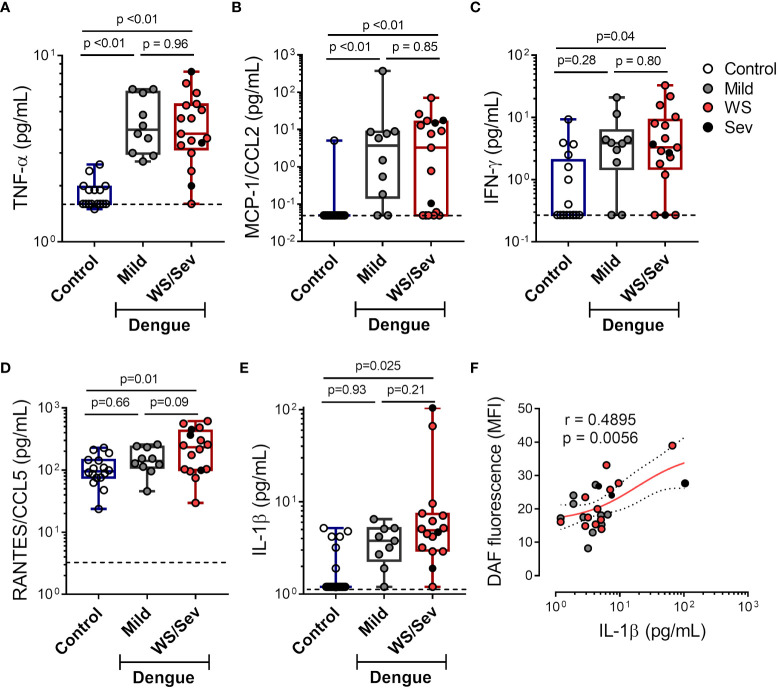
Increased levels of pro-inflammatory cytokines in plasma from dengue patients. The concentrations of **(A)** TNF-α **(B)** MCP-1/CCL2 **(C)** IFN-ɣ **(D)** RANTES/CCL5 and **(E)** IL-1β were quantified in plasma from dengue-infected patients with mild (n = 10) or that presented warning signs or severe dengue (n = 18). Each dot represents one patient or healthy volunteer. The horizontal lines in the box plots represent the median, the box edges represent the interquartile ranges, and the whiskers indicate the minimal and maximal values in each group. The dotted lines indicate the lower detection limit of each protocol. **(F)** NO synthesis in platelets was plotted against the IL-1β concentration in plasma of each dengue patient. Nonlinear regression was calculated according to the distribution of the dots.

Different proinflammatory cytokines and chemokines are known to induce iNOS expression and NO production in leukocytes and endothelial cells (8, 39–42). Hence, we quantified proinflammatory cytokines in plasma from dengue patients at acute phase. Our results demonstrate increased levels of TNF-α and MCP-1/CCL2 in patients that evolved with either mild dengue or warning signs and severe dengue syndromes, while higher levels of IFN-ɣ, RANTES/CCL5 and IL-1β were observed only in plasma from patients presenting warning signs and severe dengue (**Figures 4A-E**). Moreover, IL-1β concentration in plasma positively correlated with NO production in platelets (**Figure 4F**), suggesting a possible participation of IL-1β in platelet nitric oxide synthesis during dengue.

### IL-1β induces iNOS expression and NO synthesis in platelets

**Figure 5:**
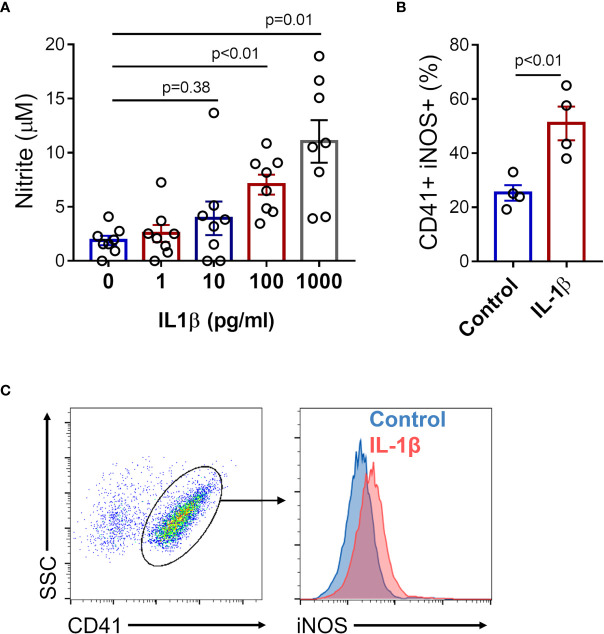
IL-1β induces iNOS expression and NO production in platelets. Platelets from healthy volunteers were stimulated with 1, 10, 100, and 1000 pg/ml of recombinant human IL-1β. **(A)** The nitrite concentration in the supernatant of platelet in each condition is shown. **(B)** The percent of intracellular iNOS expression in platelets stimulated with 1000 pg/ml of recombinant human IL-1β. The bars represent the mean ± SEM of 8 **(A)** or 4 **(B)** independent experiments with platelets from different healthy volunteers. **(C)** Gate strategy and representative histogram of one experiment is shown.

Since platelets express IL-1 receptor (IL-1R) and are capable to respond to IL-1β (43–46), we investigated whether IL-1β induces NO production in platelets. Nitrite quantification was performed in supernatants of platelets stimulated with increasing concentrations of recombinant human IL-1β, and enhanced nitrite production was observed in platelets stimulated with 100 pg/ml and 1000 pg/ml of IL-1β (**Figure 5A**). Moreover, assessment of intracellular iNOS expression by flow cytometry revealed augmented protein expression in platelets stimulated with recombinant IL-1β (1000 pg/ml) (**Figures 5B, C**).

### DENV induces platelet iNOS expression and NO production through IL-1R

**Figure 6:**
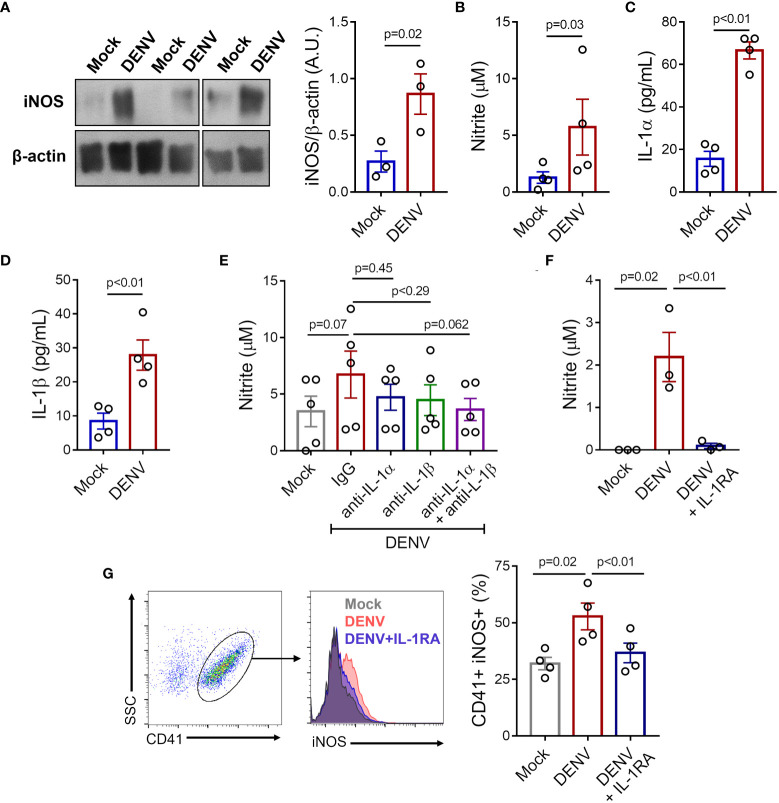
DENV induces iNOS expression and NO production in platelets through IL-1R. Platelets from healthy volunteers were stimulated with DENV-2 (MOI= 1) or Mock for 6 hours. **(A)** Expression of iNOS was analyzed by western blot in platelet lysates. β-actin was used as load control. Band densitometry of iNOS expression corrected by β-actin expression is shown (A.U., arbitrary units). **(B-D)** Quantification of **(B)** nitrite **(C)** IL-1α and **(D)** IL-1β in the supernatant of platelet in each condition. **(E-G)** Platelets from healthy volunteers were infected with DENV-2 in the presence or absence of IL-1RA, anti-IL-1α, anti-IL-β, or isotype-matched control antibody. **(E, F)** Nitrite concentration in the supernatant is shown for each condition. **(G)** The percentage of platelets with intracellular labeling for iNOS after infection with DENV in the presence or absence of IL-1RA. Representative histograms are shown. The bars represent mean ± SEM of 3 (panels **(A, F)**), 4 (panels **(B-D, G)**), or 5 (panel **(E)**) independent experiments with platelets from different healthy volunteers.

To verify if exposure to DENV *in vitro* reproduces NO synthesis as observed in platelets from patients, we incubated platelets from healthy donors with DENV-2 (MOI = 1) and evaluated iNOS expression and nitrite production. We observed that platelets exposed to DENV *in vitro* expressed higher amounts of iNOS and secreted higher levels of nitrite (**Figures 6A, B**). Importantly, platelet exposure to DENV also induced the secretion of IL-1α and IL-1β (**Figures 6C, D**).

We then investigated whether platelet IL-1-IL-1R signaling through an autocrine loop (43, 45) is involved in iNOS expression and NO synthesis in infected platelets. To gain insights in the relative roles of IL-1α and IL-1β to NO synthesis in infected platelets, we stimulated platelets with DENV in the presence of neutralizing antibodies against IL-1α and IL-1β separately and in combination. As shown in **Figure 6E**, only the blocking of both cytokines combined was able to reduce NO synthesis in platelets exposed to DENV, although only a trend towards statistical significance was observed (**Figure 6E**). To confirm the role of IL-1-IL-1R signaling, we stimulated platelets with DENV in the presence or absence of IL-1R antagonist (IL-1RA). IL-1R blocking with IL-1RA significantly prevented iNOS expression and NO production induced by DENV (**Figures 6F, G**), confirming the role of IL-1-IL-1R signaling in DENV-mediated NO synthesis in platelets.

## Discussion

It is known that platelets participate in the immune and inflammatory responses during dengue infection through interaction with leukocytes and secretion of inflammatory mediators (18, 28–31, 47, 48), including the production of NO (12, 15, 49). In the present study, we confirmed augmented production of NO by platelets during dengue infection. Even though a previous study was not able to demonstrate significant changes in NOS expression in platelets from dengue patients (12), our results show increased iNOS expression in platelets during dengue illness. Previous studies have shown NO as an inflammatory mediator with antiplatelet and anticoagulant actions and involved in endothelial dysfunction in dengue (12, 15, 17, 50). Here we show that platelet NO production in dengue correlates with disease severity markers, especially with hypoalbuminemia and haemorrhage, but not with thrombocytopenia. Our data is in agreement with higher levels of NO in association with vascular and platelet dysfunction in dengue culminating in plasma leakage and haemorrhage (14–17), and suggests platelets as a source of NO in dengue.

Here we show that soluble factors in the plasma of dengue patients contribute to the induction of iNOS and NO in platelets, and evaluated the levels of proinflammatory cytokines in patients’ plasma. Interestingly, iNOS expression was induced only by dengue patients’ plasma at higher concentration, suggesting that the mediators involved in this process have lower thresholds, since the 10-fold dilution of the plasma prevented iNOS induction. Our data show that IL-1β plasma levels was positively correlated with NO production by platelets, similarly to what has been found in macrophages from obese individuals (51). Besides IL-1β, we have also quantified IL-1α, but all patient samples presented concentrations below the detection limit (data not shown). Plasma levels of IL-1β, TNF-α and IFN-ɣ were increased in plasma from dengue patients in the present report. Despite their low levels, these cytokines are potent proinflammatory cytokines and are known to act in synergism, potentiating each other response (52, 53). Consistently, only the inhibition of IL1-α and IL-1-β toguether resulted in lower NO production in our *in vitro* infection model. Proinflammatory cytokines such as TNF-α, IFN-ɣ, and IL-1β are widely known to induce NO synthesis in different cells, including macrophages, epithelial and endothelial cells (8, 41, 42). Platelets express cytokine and chemokine receptors and respond to many of these mediators (18). TNF-α leads to platelet activation and adhesion, and contributes to the production of hyperactive platelets in a model of aging (54). Type I and type II IFNs have major roles in megakaryocyte signalling that modulate platelet production leading to thrombocytopenia and anti-viral platelet responses in many viral infections (55–58). Moreover, IL-1β stimulation changes the clotting profile and causes platelet hyperactivity (44, 46). These evidences reveal the participation of cytokines and chemokines in platelet regulation and reprograming of immune responses.

Recent studies have demonstrated important roles of IL-1β in dengue pathogenesis including in tissue injury, vascular permeability and infiltration of inflammatory cells (25, 59–61). Our group has previously demonstrated that platelet activation in dengue induces NLRP3 inflammasome activation and increased IL-1β secretion, which contributes to increased endothelial permeability (25). Furthermore, it is known that platelets express functional IL-1R (43, 44). However, whether the mechanisms regulating iNOS expression and NO synthesis by platelets involve IL-1 were not clear. Here we show that IL-1β was able to induce iNOS expression and NO production in platelets, and inhibition of IL-1-α, IL-1β and IL-1R significantly reduced virus-induced NO synthesis *in vitro*. Similarly, autocrine signaling of IL-1R has been previously shown in platelets under infectious and pro-coagulant stimulation, amplifying IL-1β secretion and integrin αIIbβ3 outside-in signaling (43, 45). Our data show that mechanisms involving platelet IL-1 secretion and engagement to IL-1R are also involved in the regulation of platelet function in dengue.

Neves-Souza and colleagues have previously shown DENV infection and iNOS expression in monocytes from dengue patients or fron DENV infection *in vitro* (62). Whether platelets are susceptible and permissive to viral infection remains a metter of controversy (63–66). A previous study has demonstrated the presence of DENV RNA and DENV-like structures within platelets from patients (67), suggesting DENV entry in platelets. Our group and others have confirmed platelets’ susceptibility to DENV infection by demonstrating that platelets can bind, internalize and support DENV genome replication and translation into viral proteins, as the number of viral RNA copies and viral proteins accumulate within platelets (29, 65, 66). However, platelets are not able to secrete new viral particles, characterizing an abortive replication (29, 68). Hence, even though platelets are susceptible to DENV infection, they are not permissive to viral replication. Although platelets cannot produce new viral particles, platelets respond to DENV infection promoting platelet activation, aggregation, and secretion of inflammatory mediators, including IL-1β (29, 66, 68). Here we confirm IL-1β and show IL-1α secretion by platelets in response to DENV *in vitro*. We also show that exposure to DENV induced iNOS expression and NO production by platelets, and highlight mechanisms of regulated iNOS expression through an IL-1-IL-1R autocrine loop. When extrapolating these results to complex systems and *in vivo* disease models we can not exclude the role of paracrine signaling in this process, since mediators present in plasma from dengue patients were also able to induce iNOS expression and NO synthesis in platelets.

This study has limitations. Since DAF-FM probe may label other nitrogen species besides NO, nitrite present in patient plasma maight have influenced platelet labeling in the plasma stimulation assay. However, exposure to dengue plasma also induced iNOS expression in platelets, highlighting the role of signaing molecules in induction of iNOS expression and NO synthesis. In our *in vitro* infection model, DENV induced platelet NO synthesis through IL-1-IL-1R engagement. Despite compeling evidence generated through pharmacological and immunological approaches, further studies using genetic models will be useful to clarify the importance of these cytokine signaling pathways in regulated expression of iNOS in platelets during dengue. Besides, considering that viremia is correlated with disease severity in dengue, titration of platelet responses to DENV *in vitro* from lower to higher MOI will be usefull to better characterize platelet-DENV interaction in the future. Even though we show the contribution of platelets as a source of NO in dengue, we cannot exclude the participation of other cells such as leukocytes and endothelial cells, as well as the participation of platelet-leukocyte and platelet-endothelial cell interactions in the modulation of NO production. Hence, further studies are required to understand the importance of platelets and other components of the immune system in pathophysiological mechanisms of dengue.

In summary, we describe novel mechanisms of platelet activation in dengue involving IL-1R-mediated NO production. Platelets from dengue patients present increased iNOS expression and NO production, which correlates with disease severity markers including hemorrhage and markers of plasma leakage. While soluble factors present in plasma are able to induce iNOS in platelets, IL-1β levels correlated with NO production by platelets in infected individuals, and IL-1β stimulation reproduced such platelet response *in vitro*. Finally, the blocking of IL-1-α, IL-1β and IL-1R prevented DENV-induced iNOS expression and NO production, demonstrating the role of an autocrine loop through IL-1R signaling in infected platelets. All these cellular events and signaling molecules are potentially involved in NO-dependent pathogenic mechanisms in dengue.

## Data availability statement

The original contributions presented in the study are included in the article. Further inquiries can be directed to the corresponding author.

## Ethics statement

The studies involving human participants were reviewed and approved by Instituto de Pesquisas Clínicas Evandro Chagas. The patients/participants provided their written informed consent to participate in this study.

## Author contributions

MP and SR performed most of the experiments, data analyses, and manuscript drafting; AQ-T and JL performed part of the experiments and data analyses; GB-L and CS performed viral propagation; FB performed clinical study design, patient inclusion, and clinical classification; PB performed clinical study design, experimental design and reviewed the manuscript; and EH performed experimental design, patient inclusion, data analysis, revised the manuscript writing and directed all aspects of the study. All authors contributed to the article and approved the submitted version.
